# Supplementary material for: Decreased BAFF Receptor Expression and Unaltered B Cell Receptor Signaling in Circulating B Cells from Primary Sjögren’s Syndrome Patients at Diagnosis
Source: Int J Mol Sci. 2022 May 4;23(9):5101. doi: 10.3390/ijms23095101 (PMC9103204; doi:10.3390/ijms23095101)
Supplement: Supplementary file 1 [file ijms-23-05101-s001.zip › ijms-1705626-SI.pdf]

International Journal of *Molecular Sciences*

MDPI

Supplementary Data To:

Decreased BAFF Receptor Expression and Unaltered B Cell Receptor Signaling in Circulating B Cells from Primary Sjögren's Syndrome Patients at Diagnosis

Stefan F. H. Neys [1], Gwenny M. Verstappen [2], Hendrika Bootsma [2], Frans G. M. Kroese [2], Rudi W. Hendriks [1,*,†] and Odilia B. J. Corneth [1,*]

Citation: Neys, S.F.H.; Verstappen, G.M.; Bootsma, H.; Kroese, F.G.M.; Hen-driks, R.W.; Corneth, O.B.J. Decreased BAFF Receptor Expression and Unaltered B Cell Receptor Signaling in Circulating B Cells from Primary Sjögren's Syndrome Patients at Diagnosis. Int. *J. Mol. Sci.* **2022**, *23*, 5101. https://doi.org/ 10.3390/ijms23095101

Academic Editor: Kenneth Michael Pollard

Received: 14 April 2022
Accepted: 30 April 2022
Published: 4 May 2022

Publisher's Note: MDPI stays neutral with regard to jurisdictional claims in published maps and institutional affiliations.

Copyright: © 2021 by the authors. Submitted for possible open access publication under the terms and conditions of the Creative Commons Attribution (CC BY) license (https://creativecommons.org/licenses/by/4.0/).

Table S1. List of antibodies and reagents used for flow cytometry staining.

Marker/Reagent	Label	Clone	Company
Human TruStain FcX	-	-	Biolegend
Fixable Viability Dye	eFluor506	-	Invitrogen
BAFFR	PerCP-Cy5.5	11C1	Biolegend
CD19	PerCP-Cy5.5	SJ25C1	BD Biosciences
CD19	FITC	HIB19	BD Biosciences
CD3	AF700	UCHT1	Invitrogen
CD3	FITC	UCHT1	Invitrogen
CD3	APC	SK7	BD Biosciences
CD3	PE	SK7	BD Biosciences
BTK	PE	53/BTK	BD phosflow
BTK	PE	REA367	Miltenyi
CD86	BV650	FUN-1	BD Biosciences
IgM	Biotin	G20-127	BD Biosciences
IgD	APC-H7	IA6-2	BD Biosciences
IgD	PE-Cy7	IA6-2	BD Biosciences
IgD	BV605	IA6-2	BD Biosciences
CD38	APC	HIT2	BD Biosciences
CD38	BV785	HIT2	Biolegend
CD27	BV421	M-T271	BD Biosciences
IgG	BV786	G-18-145	BD Biosciences
HLA-DR	BV711	G46-6	BD Biosciences
pSYK Y348	PE	I120-722	BD phosflow
pBTK Y223	AF647	N35-86	BD phosflow
pBTK Y551	PE	24a/BTK (Y551)	BD phosflow
pPLCγ2 Y759	AF647	K86-689.37	BD phosflow
Streptavidin	APC-eFluor780	-	Invitrogen
Streptavidin	BV711	-	BD Biosciences

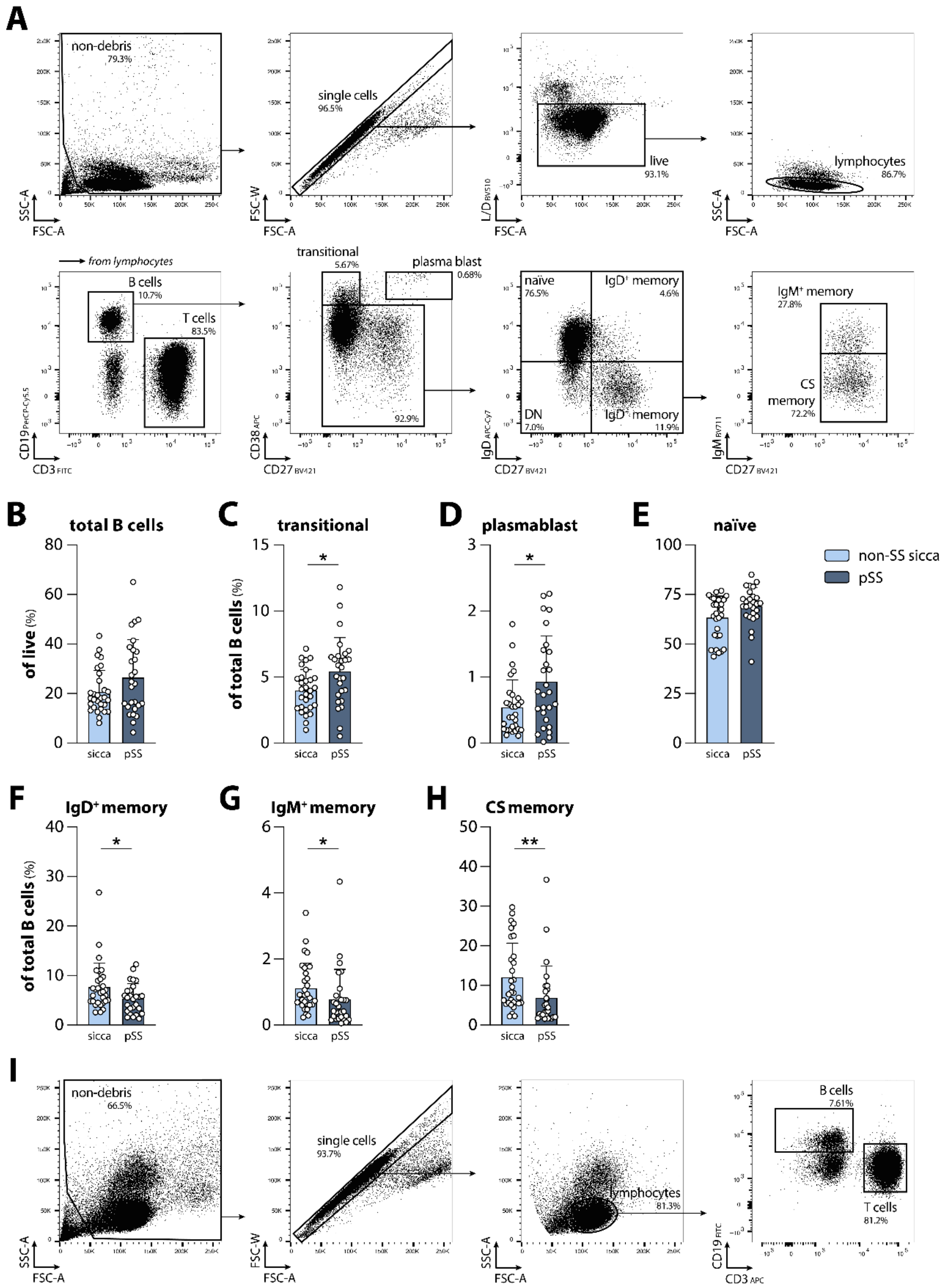

Figure S1. B cell populations in non-SS sicca and pSS patients from patient group 1. (**A**) Representative gating strategy of the intra- and extracellular marker staining. (**B**–**H**) B cell subset distribution in non-SS sicca and pSS patients, indicating (**B**) the percentage of total B cells from single, live, lymphocytes and the percentage of (**C**) transitional B cells, (**D**) plasmablasts, (**E**) naïve B cells, (**F**) IgD+ memory B cells, (**G**) IgM+ memory B cells, and (**H**) class-switched (CS) memory B cells. (**I**) Representative gating strategy of the phosphoflow cytometry staining to detect total B cells. Bars indicate mean + SD. ** $p < 0.01$, * $p < 0.05$ by unpaired t-test or Mann-Whitney U test.

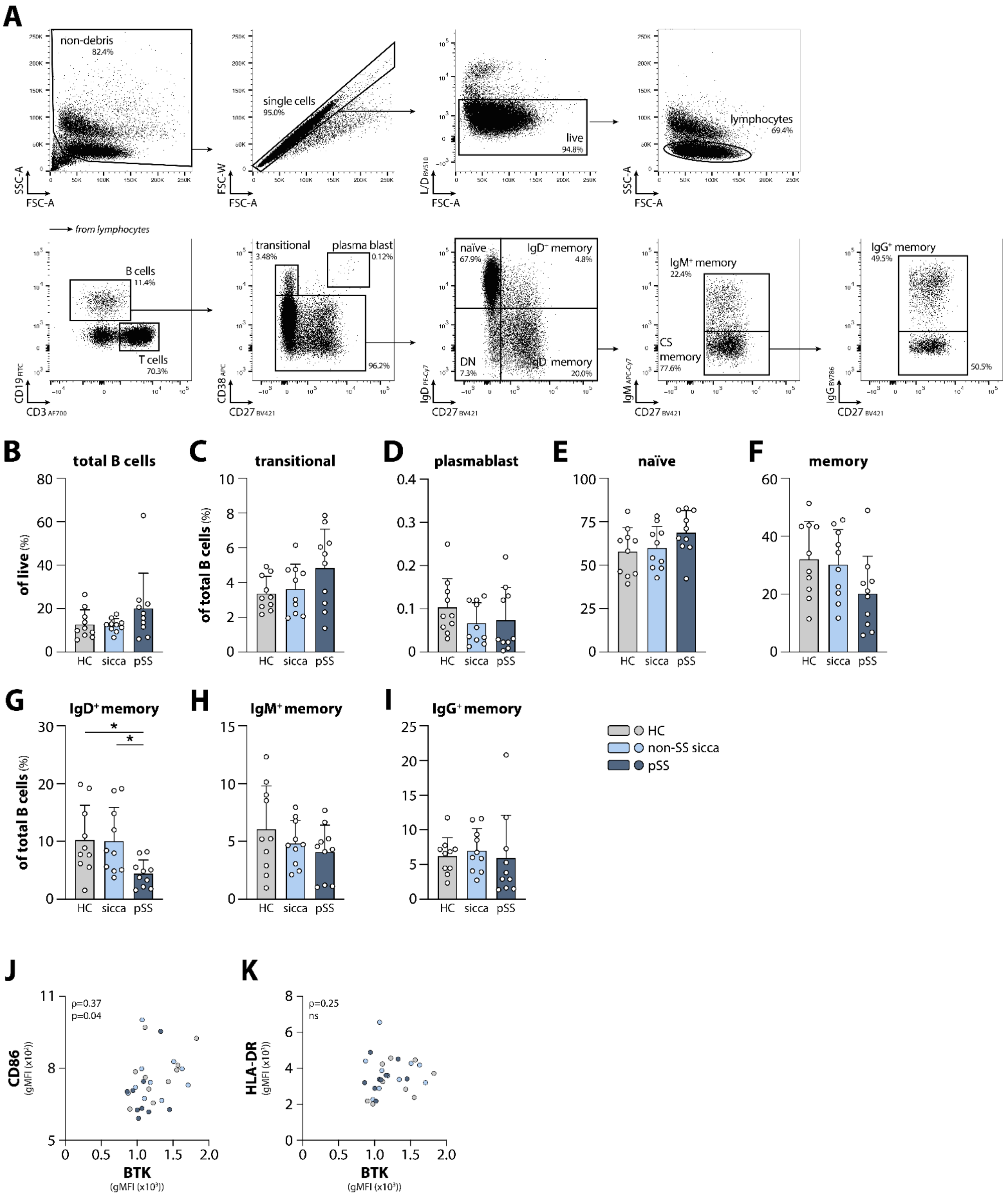

Figure S2. B cell population analysis of HCs, non-SS sicca patients, and pSS patients from patient group 2. (**A**) Representative gating strategy of the intra- and extracellular marker staining. (**B–I**) B cell subset distribution in HC, non-SS sicca patients, and pSS patients, with (**B**) the percentage of total B cells from single, live, lymphocytes and the percentage of (**C**) transitional B cells, (**D**) plasmablasts, (**E**) naïve B cells, (**F**) total memory B cells, (**G**) IgD^{+} memory B cells, (**H**) IgM^{+} memory B cells, and (**I**) IgG^{+} memory B cells. Bars indicate mean + SD. (**J**, **K**) Spearman's rank correlation analysis between intracellular BTK expression in total B cells and (**J**) surface CD86 and (**K**) surface HLA-DR expression. * $p < 0.05$ by Kruskal-Wallis test with Dunn's correction for multiple comparisons.

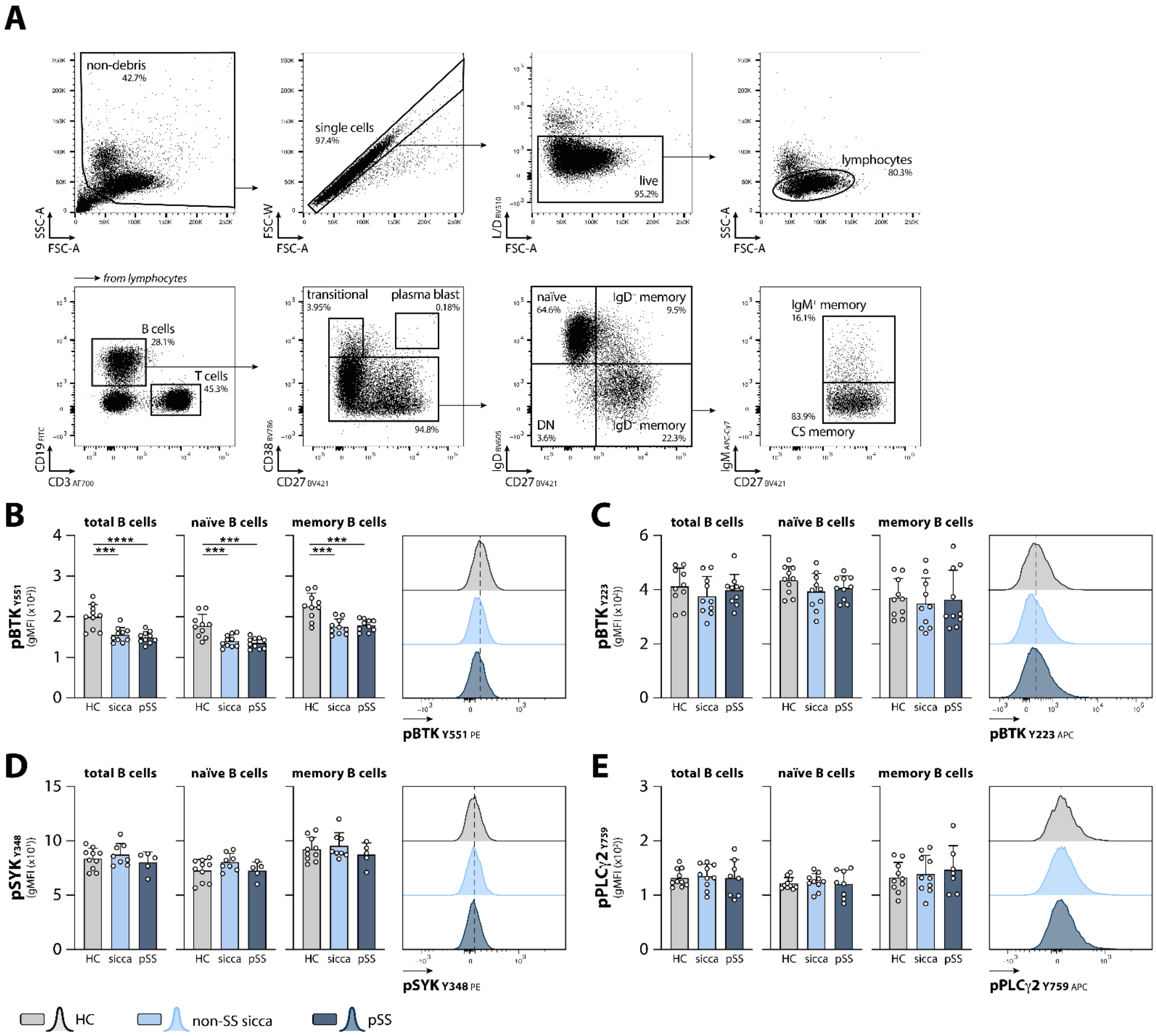

Figure S3. Basal BCR signaling activity in HCs, non-SS sicca patients, and pSS patients. (**A**) Representative gating strategy of the intra- and extracellular marker staining. (**B–E**) Basal phosphorylation levels of (**B**) BTK (Y551), (**C**) BTK (Y223), (**D**) SYK (Y348), and (**E**) PLCγ2 (Y759) in circulating total, naïve, and memory B cells in HCs, non-SS sicca patients, and pSS patients. Representative histograms are shown for total B cells. Bars indicate mean + SD. **** $p < 0.0001$, *** $p < 0.001$ by Kruskal-Wallis test with Dunn's correction for multiple comparisons.
